# Influenza, COVID-19, and pneumococcal vaccination status among adults in Türkiye

**DOI:** 10.55730/1300-0144.6368

**Published:** 2026-05-06

**Authors:** Gamze ŞANLIDAĞ İŞBİLEN, Serap GENÇER, Hüsnü PULLUKÇU, Meltem TAŞBAKAN, İftahar KÖKSAL

**Affiliations:** 1 Department of Infectious Diseases and Clinical Microbiology, Sancaktepe Şehit Prof. Dr. İlhan Varank Training and Research Hospital, İstanbul, Turkiye; 2 Department of Infectious Diseases and Clinical Microbiology, Faculty of Medicine, Ege University, İzmir, Turkiye; 3 Department of Infectious Diseases and Clinical Microbiology, Faculty of Medicine, Acıbadem University, İstanbul, Turkiye; 4 Turkish Society of Clinical Microbiology and Infectious Diseases (EKMUD) Immunization Working Group, Ankara, Turkiye

**Keywords:** COVID-19, influenza, pneumococcal, vaccine

## Abstract

**Background/aim:**

Vaccines reduce morbidity and mortality caused by infectious diseases that threaten public health. Lifelong immunization programs are essential for the entire population. This study assessed adult vaccination uptake for influenza, pneumococcal, and coronavirus disease 2019 (COVID-19) vaccines in Türkiye and examined the barriers and factors influencing vaccination decisions.

**Materials and methods:**

This cross-sectional survey was designed and conducted by the Immunization Working Group of the Turkish Society of Clinical Microbiology and Infectious Diseases. A 19-item questionnaire was administered to individuals aged 18 years or older to assess influenza, pneumococcal, and COVID-19 vaccination status. The questionnaire collected data on demographic characteristics, healthcare worker (HCW) status, chronic diseases, vaccination history, vaccine preferences, and reasons for nonvaccination.

**Results:**

The influenza vaccination rate among all participants was 28%, with the highest rate observed among participants aged ≥65 years (49%). Multivariate analysis revealed that age ≥65 years, HCW status, and the presence of a chronic disease were independently associated with influenza vaccination (p < 0.001). Pneumococcal vaccination rates were higher among individuals with chronic diseases and those aged ≥65 years (p < 0.01). Among chronic diseases, the highest vaccination rate was observed in participants with coronary artery disease. The COVID-19 vaccination rate was 95%. The most common reasons for nonvaccination were the perception that influenza (39%) and pneumococcal (21%) vaccination was unnecessary, whereas unwillingness to be vaccinated (44%) and concerns about side effects (32%) predominated for COVID-19 vaccination.

**Conclusions:**

Vaccination coverage remains low in Türkiye, even among high-risk groups. Although vaccination rates increased during the pandemic, greater public awareness and educational efforts are still needed.

## 1. Introduction

Vaccines represent one of the most significant advancements in medical history. They have reduced morbidity and mortality caused by infectious diseases that threaten public health [1]. The World Health Organization (WHO) estimates that immunization prevents 3.5 to 5 million deaths annually^1^. Vaccination programs provide direct protection to those vaccinated. They also contribute to herd immunity by increasing vaccination coverage, slowing disease transmission, and reducing the risk of infection among vulnerable populations [2].

Childhood immunization programs have demonstrated remarkable success, substantially reducing the incidence of vaccine-preventable diseases^2^. In contrast, adult immunization programs have not yet achieved comparable levels of success [3,4]. Current adult vaccination strategies prioritize immunocompromised individuals and those with chronic conditions [3]. However, limiting vaccination to these specific groups is insufficient to achieve population-level immunity because disease risks persist within these populations^2^. Therefore, comprehensive lifelong immunization strategies that encompass the entire population, rather than only targeted groups, remain essential [5]. The WHO’s Immunization Agenda 2030 envisions a world in which everyone, regardless of age or location, benefits from vaccines, thereby improving overall health outcomes^3^.

This study aimed to assess the uptake of influenza, pneumococcal, and coronavirus disease 2019 (COVID-19) vaccines among adults in Türkiye. It also examined the reasons for nonvaccination and the factors influencing individuals’ willingness to be vaccinated.

## 2. Materials and methods

This cross-sectional survey, designed by the Immunization Working Group of the Turkish Society of Clinical Microbiology and Infectious Diseases (EKMUD), used a 19-item questionnaire to assess the uptake of influenza, pneumococcal, and COVID-19 vaccines among adults in Türkiye. The survey was conducted between 1 October 2023, and 1 October 2024, through online platforms and face-to-face interviews and targeted adults aged ≥18 years from diverse backgrounds. The questionnaire was distributed through the nationwide network of infectious disease specialists and clinical microbiologists affiliated with the EKMUD Immunization Working Group. Clinicians participating in the online survey system were encouraged to conduct face-to-face interviews; however, the identities of the participating centers were not recorded, thereby ensuring institution-independent data collection. To prevent duplicate responses and ensure data integrity, the online survey permitted only one submission per device through IP address restrictions, and face-to-face participants were asked whether they had previously completed the survey. The face-to-face questionnaires were administered by infectious disease and clinical microbiology specialists who were members of the EKMUD Immunization Working Group, and the responses were entered into the study database by the authors. No training was provided before the questionnaires were administered. Participation was voluntary and anonymous.

The questionnaire collected data on participants’ age, sex, occupation, and educational level. The questionnaire also collected data on healthcare worker status, the presence of chronic diseases for which vaccination is recommended (e.g., diabetes mellitus, malignancy, rheumatologic disease, immunodeficiency, and chronic pulmonary disease), and vaccination status for COVID-19, influenza, and pneumococcal disease. Additional information included vaccine preferences, vaccination timing, and reasons for nonvaccination, where applicable. Clinical conditions for which pneumococcal vaccination is recommended were evaluated. These conditions included diabetes mellitus, malignancy, rheumatologic diseases, coronary artery disease, immunodeficiencies (including human immunodeficiency virus [HIV] infection and common variable immunodeficiency), chronic pulmonary disease, chronic kidney disease, chronic liver disease, and inflammatory bowel disease.

Ethical approval was obtained from the Acıbadem University Medical Research Ethics Committee (approval no. 2023-11/37).

Data were analyzed using IBM SPSS Statistics, version 22.0 (IBM Corp., Armonk, NY, USA). Categorical variables were summarized as frequencies and percentages, whereas continuous variables were summarized as means and standard deviations or medians and interquartile ranges (IQRs), as appropriate.

For between-group comparisons, the chi-square test was used for categorical variables, whereas the independent-samples t-test was used for continuous variables. Univariate logistic regression analysis was performed to assess factors associated with influenza, pneumococcal, and COVID-19 vaccination status. Variables that were significant in the univariate analysis (age, educational level, healthcare worker status, and the presence of chronic diseases for which vaccination is recommended), together with clinically relevant variables, were included in the multivariable logistic regression model using the Enter method. The significance of the model was evaluated using the omnibus test, and model fit was assessed with the Hosmer–Lemeshow goodness-of-fit test. The model’s explanatory power was assessed using the Cox and Snell R^2^ and Nagelkerke R^2^ statistics, whereas classification performance was evaluated based on the overall classification accuracy. Multivariate analysis results were presented as adjusted odds ratios (aOR) with 95% confidence intervals (CI).

A two-sided p < 0.05 was considered statistically significant. For each vaccine, responses of “cannot recall” were treated as missing data and were excluded from the analysis for the corresponding question; therefore, percentages were calculated using only valid responses.

## 3. Results

### 3.1. Participant characteristics

A total of 4279 participants were included in the study. The mean age of the participants was 43.12 ± 14.57 years (median: 44 years; IQR: 30–55), and 2777 (65%) were female. Among the participants, 266 individuals (6.2%) were aged 65 years or older. A total of 2047 participants (47.8%) were healthcare workers. The most frequently reported chronic conditions were hypertension (n = 647, 15%), thyroid disorders (n = 511, 12%), and diabetes mellitus (n = 311, 7%). Of the participants, 903 (21%) had an indication for pneumococcal vaccination due to chronic diseases. The demographic and clinical characteristics of the participants are presented in Table 1.

Of the 4279 questionnaires, 95 (2.2%) were administered face-to-face, whereas 4184 (97.8%) were completed online. Because of the substantial imbalance in group sizes, no statistical comparison was performed between the two survey modalities.

### 3.2. Influenza vaccination

The overall influenza vaccination rate among all participants was 28% (n = 1122). Among vaccinated participants, 648 (62%) had received their most recent influenza vaccination within the preceding year. A total of 2908 participants (72%) had not received the influenza vaccine. Participants who answered “cannot recall” to this question (n = 249) were excluded from the analysis.

In the univariate analysis (Table 2), the influenza vaccination rate was significantly higher among participants aged *≥*65 years (49%) than among those aged <65 years (26%) (odds ratio [OR], 2.73; 95% CI, 2.12–3.52; p < 0.001). Healthcare workers had a significantly higher influenza vaccination rate (33%) than nonhealthcare workers (23%) (OR, 1.61; 95% CI, 1.40–1.86; p < 0.001). The influenza vaccination rate was also significantly higher among participants with chronic conditions for which vaccination is recommended (37%) than among those without such conditions (25%) (OR, 1.72; 95% CI, 1.47–2.02; p < 0.001). Regarding educational level, the influenza vaccination rate was significantly higher among participants with postgraduate education (33%) than among those with primary, secondary or, high school education (27%) (OR, 1.28; 95% CI, 1.03–1.60; p = 0.028). No significant difference was observed between sexes (28% among females versus 28% among males; p = 0.822).

After adjustment for potential confounding factors using multivariable logistic regression analysis (Table 3), age *≥*65 years (aOR, 2.66; 95% CI, 2.03–3.49; p < 0.001), healthcare worker status (aOR, 1.75; 95% CI, 1.49–2.05; p < 0.001), and the presence of chronic conditions for which vaccination is recommended (aOR, 1.56; 95% CI, 1.32–1.85; p < 0.001) were independently associated with influenza vaccination. Educational level was not independently associated with influenza vaccination in the multivariable analysis (aOR for postgraduate education, 1.13; 95% CI, 0.87–1.45; p = 0.363). The model’s overall classification accuracy was 73%.

Among participants with chronic conditions included in this analysis (coronary artery disease, malignancy, chronic pulmonary disease, diabetes mellitus, and rheumatologic disease), the influenza vaccination rate was 36.9%. Statistical analysis was not performed for other chronic diseases due to the small number of patients. When examined by these specific diseases, the rates were as follows: coronary artery disease (43.2%), malignancy (41.6%), chronic pulmonary disease (38.8%), diabetes mellitus (38.3%), and rheumatologic disease (28.8%). Across all of these subgroups, most vaccinated participants had received their influenza vaccine within the preceding year. Among participants with chronic conditions, no significant difference in influenza vaccination rates was observed between females and males (36.3% versus 38%; p = 0.629).

Among participants aged ≥65 years, influenza vaccination rates were significantly higher in those with chronic conditions than in those without chronic conditions (56.3% versus 43.8%; p = 0.043).

### 3.3. Pneumococcal vaccination

A total of 954 eligible participants (aged ≥65 years or with chronic conditions indicating pneumococcal vaccination) were included in the analysis. At the time of the study, the Centers for Disease Control and Prevention (CDC) recommended pneumococcal vaccination for adults aged ≥65 years. In October 2024, these recommendations were updated to include adults aged ≥50 years^4^. In this group, 35% (n = 338) had received pneumococcal vaccination. Among participants with available information on vaccine type, the 13-valent pneumococcal conjugate vaccine (PCV13) was the most frequently administered vaccine (59%).

In the univariate analysis (Table 4), the mean age of vaccinated participants was 57 ± 14 years. This was significantly higher than the mean age of unvaccinated participants (52 ± 13 years; p < 0.001). The vaccination rate among individuals with coronary artery disease was 50%, compared to 37% for those without (OR, 1.74; 95% CI, 1.13–2.67; p = 0.010). Pneumococcal vaccination rates were 37% among females and 39% among males, with no statistically significant difference between the groups (p = 0.532). Pneumococcal vaccination was more common among participants with postgraduate education (41%) than among those with primary, secondary, or high school education (36%); however, this difference was not statistically significant (p = 0.210). No significant associations were observed between pneumococcal vaccination status and diabetes mellitus, chronic pulmonary disease, rheumatologic disease, or malignancy (all p > 0.05).

When the analysis was restricted to specific chronic conditions, pneumococcal vaccination rates were highest among participants with coronary artery disease (50%), followed by chronic pulmonary disease (43.2%), diabetes mellitus (40.2%), malignancy (39.2%), and rheumatologic disease (32.4%). Among participants with chronic conditions, pneumococcal vaccination rates were 37% among females and 39.9% among males; this difference was not statistically significant (p = 0.385).

Among participants aged ≥65 years, a group at high risk for invasive pneumococcal disease, the pneumococcal vaccination rate was 52%. Only 16.4% of these participants had received both a pneumococcal conjugate vaccine and a pneumococcal polysaccharide vaccine. Additionally, 63.3% of participants could not recall which type of pneumococcal vaccine they had received. Additionally, 71.4% of participants in this group reported having received only a single dose. Most participants reported receiving only a single vaccine dose. Pneumococcal vaccination rates were 54.2% among females and 49% among males, with no statistically significant difference between the groups (p = 0.532).

Among participants aged ≥65 years, pneumococcal vaccination rates by educational level were as follows: postgraduate education (54.5%), university education (50.8%), primary school education (41.9%), and middle/high school education (39.3%). These differences were not statistically significant (p = 0.322).

Among participants aged ≥65 years, pneumococcal vaccination rates were significantly higher in those with chronic conditions than in those without chronic conditions (57.2% versus 35.6%; p = 0.04). Similarly, among participants with chronic conditions, pneumococcal vaccination rates were significantly higher in those aged ≥65 years than in those aged <65 years (57.2% versus 32.9%; p < 0.001).

### 3.4. COVID-19 vaccination

The overall COVID-19 vaccination rate among participants was 95%. The most commonly received vaccine was the mRNA vaccine tozinameran (Comirnaty; Pfizer-BioNTech, Mainz, Germany), followed by the inactivated vaccines CoronaVac (Sinovac Biotech, Beijing, China) and TurkoVac (TÜSEB, Ankara, Türkiye). Among vaccinated participants, 3292 (90%) had received their most recent COVID-19 vaccine dose more than 12 months before the survey. A three-dose regimen was the most frequently reported (35%). Participants who responded “cannot recall” to the COVID-19 vaccination question (n = 11) were excluded from the COVID-19 vaccine-related analyses.

In the univariate analysis (Table 5), the COVID-19 vaccination rate was significantly higher among participants with postgraduate education (97%) than among those with primary, secondary, or high school education (94%) (OR, 1.93; 95% CI, 1.24–3.01; p = 0.004). Healthcare workers had a significantly higher COVID-19 vaccination rate (97%) than nonhealthcare workers (94%) (OR, 1.70; 95% CI, 1.27–2.28; p < 0.001). Although the COVID-19 vaccination rate was higher among participants aged *≥*65 years (97%) than among those aged <65 years (95%), the difference was not statistically significant (p = 0.179). No significant associations were observed between COVID-19 vaccination status and sex, the presence of chronic conditions, diabetes mellitus, chronic pulmonary disease, rheumatologic disease, malignancy, or coronary artery disease (all p > 0.05).

### 3.5. Vaccine hesitancy

The reasons for nonvaccination are presented in Table 6. For influenza vaccination, the most frequently reported reasons for nonvaccination in the general population were the perception that vaccination was unnecessary (39%), unwillingness to be vaccinated (21%), concerns about side effects (12%), and lack of information about the vaccine (12%). Among healthcare workers, a priority group for influenza vaccination, the perception that vaccination was unnecessary remained the most frequently reported reason for nonvaccination.

When analyzed by educational level, lack of knowledge about the vaccine was the most frequently reported reason for nonvaccination among participants with only primary school education. In contrast, among participants in the other educational groups, the most frequently reported reason was the perception that vaccination was unnecessary.

Among participants aged ≥65 years, who are at high risk for influenza-related complications, the most frequently reported reason for nonvaccination was the perception that vaccination was unnecessary, consistent with the findings in younger age groups.

For pneumococcal vaccination, the most frequently reported reasons for nonvaccination were the perception that vaccination was unnecessary or the absence of a perceived medical indication for vaccination (combined, 41%), insufficient information about the vaccine, including a lack of physician recommendation and patient’s lack of information (33%), and unwillingness to be vaccinated (13%).

Despite being at high risk for invasive pneumococcal disease, participants aged ≥65 years most frequently reported the perception that vaccination was unnecessary, consistent with the findings in the younger age groups.

Although the COVID-19 vaccination rate was high, the most frequently reported reasons for nonvaccination were unwillingness to be vaccinated (44%), concerns about previous side effects (32%), and the perception that vaccination was unnecessary (28%).

## 4. Discussion

This study highlights that influenza, pneumococcal, and COVID-19 vaccination coverage remains low in Türkiye, including among populations at the highest risk, despite these vaccines being essential components of adult immunization programs.

According to the WHO, approximately one billion cases of influenza occur worldwide each year, of which 3 to 5 million are severe, resulting in an estimated 290,000 to 650,000 deaths^5^. Data from the CDC indicate that influenza vaccination substantially reduces influenza-related hospitalizations and mortality [6,7]. Vaccination also remains one of the most cost-effective interventions for preventing influenza [8,9]. Despite this established efficacy, our analysis shows that influenza vaccination rates remain suboptimal.

Although healthcare workers had higher influenza vaccination rates than the general population, these rates remained lower than those reported in comparable studies. Previous research has identified variations by professional category; however, our study did not categorize healthcare workers by profession, preventing such analysis [10–12]. Furthermore, higher educational level was associated with higher influenza vaccination rates, consistent with previous studies [13–15]. This finding suggests that greater awareness may contribute to higher vaccination uptake among individuals with higher educational attainment.

Influenza and pneumococcal vaccination rates were higher among participants aged ≥65 years than among other risk groups and were also higher than those reported in previous studies [16–18]. This finding may reflect the impact of public health initiatives and more frequent contact with healthcare providers among older adults.

Across all groups, the predominant reason for influenza vaccine hesitancy was the perception that vaccination is unnecessary, which corresponds with existing literature [19]. This belief may stem from underestimating disease severity or failing to identify oneself as belonging to a risk category.

Consistent with previous research, influenza vaccination rates were higher than pneumococcal vaccination rates among participants for whom pneumococcal vaccination was recommended [18–20]. Among participants who had received only a single dose, the proportions were similar for influenza and pneumococcal vaccination. One possible explanation is that pneumococcal vaccination schedules may not always be completed in clinical practice. Although pneumococcal vaccination rates have improved compared with previous years, they remain suboptimal [21].

Among participants with chronic conditions, those with chronic pulmonary disease had the highest pneumococcal vaccination rate, which was higher than that reported in previous studies from Türkiye. Nevertheless, consistent with previous research, insufficient physician recommendation remained the most frequently reported reason for pneumococcal vaccine nonuptake [22]. Despite the availability of national and international guidelines, strategies to improve physician recommendations and patient education should be further evaluated and strengthened. Clinicians should be encouraged to discuss vaccination proactively with eligible patients, and healthcare systems should implement targeted educational initiatives to improve vaccination uptake^2^.

Research conducted in clinics specializing in chronic disease management has demonstrated higher vaccination rates [23]. Similarly, a study conducted in cardiology outpatient clinics in Türkiye reported higher pneumococcal vaccination rates than those observed in our study and emphasized the important role of healthcare professionals in promoting vaccination [24]. The enhanced awareness among healthcare providers in these specialized settings, along with their increased propensity to educate patients, may contribute to improved vaccination rates. Studies documenting lower rates in previous years also highlight the positive impact of recent initiatives [22,25].

The complexity of pneumococcal vaccination protocols based on chronic conditions and vaccination history has been identified as an implementation barrier [26]. The recent introduction of the 20-valent pneumococcal conjugate vaccine in Türkiye—which can be administered as a single dose to previously unvaccinated individuals or following the 13-valent pneumococcal conjugate vaccine—may simplify vaccination recommendations and facilitate implementation.

COVID-19 caused a pandemic associated with substantial mortality; however, mortality declined rapidly after the introduction of vaccines [27]. The COVID-19 vaccination rate observed in our study was higher than the reported national average^6^. This may be attributed to the fact that approximately half of the participants were healthcare professionals. Earlier research conducted at the onset of the pandemic, before vaccine availability, documented higher levels of vaccine hesitancy [28].

One of the strengths of this study is its large sample size, achieved by recruiting participants from diverse adult populations across Türkiye. Furthermore, the use of both face-to-face interviews and online surveys increased the diversity of participants. The combined assessment of influenza, pneumococcal, and COVID-19 vaccination provided valuable insights into adult vaccination uptake. Multivariable analysis identified several factors that were independently associated with vaccination uptake.

Despite these strengths, this study has several limitations. The cross-sectional design allowed the identification of associations but did not permit causal inferences. Additionally, ease of use and the use of online platforms may have led to selection bias. For example, the high proportion of healthcare workers in the study may limit the generalizability of the findings to the broader adult population. Because data were collected using both online and face-to-face surveys, the potential influence of response biases, including social desirability bias, nonresponse bias, and acquiescence bias, could not be fully assessed. Finally, the use of self-reported data may have introduced recall bias, particularly regarding specific vaccine types or the timing of previous doses. This limitation is reflected in the exclusion of responses from participants who were unable to recall their vaccination history.

In conclusion, influenza and pneumococcal vaccination rates remain below target levels. Although coverage among priority populations has improved compared to previous periods, it remains insufficient. Strengthening collaboration with physicians who provide care for these populations may help improve vaccination rates. The suboptimal vaccination rates observed even among healthcare workers, who play a key role in promoting vaccination, highlight the need for interventions specifically targeting this population. Although COVID-19 vaccination coverage was substantial during the pandemic period, individualized assessments and evaluation of booster doses are necessary.

## Figures and Tables

**Table 1:** Demographic characteristics of the study participants.

Variable	n (%)
*Sex*	
Female	2777 (64.9%)
Male	1502 (35.1%)
*Age group*	
18–29 years	1045 (24.4%)
30–49 years	1655 (38.7%)
50–64 years	1313 (30.7%)
≥65 years	266 (6.2%)
*Education level*	
Primary, secondary, or high school	583 (13.6%)
University	2344 (54.8%)
Master’s or doctoral degree	1352 (31.6%)
*Chronic conditions*	
Hypertension*	647 (15%)
Thyroid disorders*	511 (12.0%)
Diabetes mellitus	311 (7.3%)
Chronic pulmonary disease	262 (6%)
Rheumatologic disease	186 (4.3%)
Malignancy	115 (2.7%)
Coronary artery disease	99 (2.3%)
Other (CKD, HIV, IBD, MS, CLD)†	169 (4.0%)
*Healthcare worker*	
Yes	2047 (47.8%)
No	2232 (52.2%)

* Hypertension and thyroid disorders are not considered conditions for which vaccination is recommended and were therefore excluded from the analyses of vaccination eligibility.

† CKD: chronic kidney disease; HIV: human immunodeficiency virus; IBD: inflammatory bowel disease; MS: multiple sclerosis; CLD: chronic liver disease.

**Table 2:** Univariable analysis of factors associated with influenza vaccination among the study participants (n = 4030).

Variable	Category	Vaccinated n (%)	Not vaccinated n (%)	OR (95% CI)	p
Age group	≥65 years	130 (12%)	133 (5%)	2.73 (2.12–3.52)	**<0.001**
	<65 years	992 (88%)	2775 (95%)		
Sex	Female	740 (66%)	1907 (66%)	1.02 (0.88–1.18)	0.822
	Male	382 (34%)	1001 (34%)		
Education level	Master’s or doctoral degree	429 (38%)	891 (31%)	1.28 (1.03–1.60)	**0.028**
	University degree	545 (49%)	1623 (56%)	0.89 (0.72–1.11)	0.301
	Primary, secondary, or high school	148 (13%)	394 (14%)	1.00	-
Healthcare worker status	Healthcare worker	647 (58%)	1331 (46%)	1.61 (1.40–1.86)	**<0.001**
	Nonhealthcare worker	475 (42%)	1577 (54%)		
Presence of a chronic condition for which vaccination is recommended	Yes	323 (29%)	553 (19%)	1.72 (1.47–2.02)	**<0.001**
	No	799 (71%)	2355 (81%)		

Percentages were calculated using valid cases only (n = 4030). Crude odds ratios (ORs) and 95% confidence intervals (CIs) were estimated using univariable logistic regression for each independent variable. For educational level, ORs and p-values were obtained from a logistic regression model including educational categories only. Bold p-values indicate statistical significance (p < 0.05). Categorical variables were compared using the chi-square test.

**Table 3:** Multivariable logistic regression analysis of factors associated with influenza vaccination among the study participants (n = 4030).

Variable	Category	B	SE	Wald	aOR (Exp(B))	95% CI for aOR	p-value
Age group	≥65 years	0.978	0.139	49.59	2.66	2.03–3.49	**<0.001**
Education level	Master’s or doctoral degree	0.117	0.129	0.83	1.13	0.87–1.45	0.363
	University degree	−0.080	0.116	0.47	0.92	0.74–1.16	0.493
Healthcare worker status	Yes	0.558	0.081	46.96	1.75	1.49–2.05	**<0.001**
Presence of a chronic condition for which vaccination is recommended	Yes	0.446	0.087	26.50	1.56	1.32–1.85	**<0.001**

Adjusted odds ratios (aORs) and 95% confidence intervals (CIs) were estimated using a multivariable logistic regression model that included all variables shown (enter method).

Model fit statistics: omnibus chi-square test, χ^2^(6) = 173.51, p < 0.001 (shown in bold); −2 log likelihood = 4593.56; Cox and Snell R^2^ = 0.042; Nagelkerke R^2^ = 0.061; Hosmer–Lemeshow goodness-of-fit test, χ^2^(6) = 6.15, p = 0.41; overall classification accuracy = 72.5%.

**Table 4:** Univariable analysis of factors associated with pneumococcal vaccination among adults for whom pneumococcal vaccination is recommended (n = 954).

Variable	Category	Vaccinated n (%)	Not vaccinated n (%)	OR (95% CI)	p
Age (years)^1^	-	57 ± 14	52 ± 13	-	**<0.001**
Sex	Female	229 (63%)	388 (65%)	0.92 (0.70–1.20)	0.532
	Male	132 (37%)	205 (35%)		
Education level	Master’s or doctoral degree	118 (33%)	168 (28%)	1.27 (0.87–1.85)	0.210
	University degree	174 (48%)	300 (51%)	1.05 (0.74–1.49)	0.781
	Primary, secondary, or high school	69 (19%)	125 (21%)	1.00	-
Presence of a chronic condition for which vaccination is recommended	Yes	302 (84%)	517 (87%)	0.75 (0.52–1.09)	0.130
	No	59 (16%)	76 (13%)		
Diabetes mellitus	Yes	115 (32%)	171 (29%)	1.15 (0.87–1.53)	0.324
	No	246 (68%)	422 (71%)		
Chronic pulmonary disease	Yes	101 (28%)	133 (22%)	1.34 (1.00–1.81)	0.053
	No	260 (72%)	460 (78%)		
Rheumatologic disease	Yes	56 (16%)	117 (20%)	0.75 (0.53–1.06)	0.101
	No	305 (84%)	476 (80%)		
Malignancy	Yes	40 (11%)	62 (10%)	1.07 (0.70–1.63)	0.762
	No	321 (89%)	531 (90%)		
Coronary artery disease	Yes	47 (13%)	47 (8%)	1.74 (1.13–2.67)	**0.010**
	No	314 (87%)	546 (92%)		
Other chronic conditions	Yes	51 (14%)	69 (12%)	1.25 (0.85–1.84)	0.260
	No	310 (86%)	524 (88%)		

1 The study population for this analysis consisted of adults aged ≥65 years or adults aged 18–64 years with at least one chronic condition for which pneumococcal vaccination is recommended. Age is presented as mean ± standard deviation (SD). The p-value for age was obtained using an independent-samples t test (equal variances assumed). Categorical variables were compared between vaccinated and unvaccinated participants using the chi-square test. Crude odds ratios (ORs) and 95% confidence intervals (CIs) were estimated using univariable logistic regression or 2 × 2 risk estimates, as appropriate. For educational level, ORs and p-values were obtained from a separate logistic regression model including educational categories only, with primary–secondary–high school as the reference category. Bold p-values indicate statistical significance (p < 0.05).

**Table 5:** Univariable analysis of factors associated with COVID-19 vaccination among the study participants (n = 4268).

Variable	Category	Vaccinated n (%)	Not vaccinated n (%)	OR (95% CI)	p
Age group	≥65 years	256 (6%)	8 (4%)	1.63 (0.79–3.34)	0.179
	<65 years	3810 (94%)	194 (96%)		
Sex	Female	2641 (65%)	130 (64%)	1.03 (0.76–1.38)	0.862
	Male	1425 (35%)	72 (36%)		
Education level	Master’s or doctoral degree	1305 (32%)	46 (23%)	1.93 (1.24–3.01)	**0.004**
	University degree	2218 (55%)	119 (59%)	1.27 (0.87–1.86)	0.218
	Primary, secondary, or high school	543 (13%)	37 (18%)	1.00	-
Healthcare worker status	Healthcare worker	1971 (49%)	72 (36%)	1.70 (1.27–2.28)	**<0.001**
	Nonhealthcare worker	2095 (52%)	130 (64%)		
Diabetes mellitus	Yes	298 (7%)	13 (6%)	1.15 (0.65–2.04)	0.633
	No	3768 (93%)	189 (94%)		
Chronic pulmonary disease	Yes	249 (6%)	11 (5%)	1.13 (0.61–2.11)	0.694
	No	3817 (94%)	191 (95%)		
Rheumatologic disease	Yes	176 (4%)	10 (5%)	0.87 (0.45–1.67)	0.673
	No	3890 (96%)	192 (95%)		
Malignancy	Yes	110 (3%)	5 (2%)	1.10 (0.44–2.72)	0.844
	No	3956 (97%)	197 (98%)		
Coronary artery disease	Yes	90 (2%)	8 (4%)	0.55 (0.26–1.15)	0.106
	No	3976 (98%)	194 (96%)		
Other chronic conditions	Yes	151 (4%)	7 (3%)	1.07 (0.50–2.32)	0.855
	No	3915 (96%)	195 (97%)		

Percentages are column percentages (within COVID-19 vaccination status) and are based on valid cases only (n = 4268). Categorical variables were compared using the chi-square test. Odds ratios (ORs) and 95% confidence intervals (CIs) were estimated using univariable logistic regression or 2 × 2 risk estimates, as appropriate. For educational level, ORs and p-values were obtained from a separate logistic regression model including educational categories only, with primary–secondary–high school as the reference category. Bold p-values indicate statistical significance (p < 0.05).

**Table 6:** Reasons for nonvaccination against influenza, pneumococcal disease, and COVID-19 (%).

Reason for not receiving vaccination	Influenza n (%) (n = 2908)	Pneumococcal n (%) (n = 593)	COVID-19 n (%) (n = 202)
Could not find/access the vaccine	286 (10%)	27 (5%)	6 (3%)
Lack of information about the vaccine	343 (12%)	124 (21%)	7 (4%)
Concerned about side effects	350 (12%)	51 (9%)	64 (32%)
Previously experienced side effects	130 (4%)	9 (2%)	5 (3%)
Did not consider vaccination necessary	1120 (39%)	126 (21%)	57 (28%)
Physician did not recommend vaccination	161 (6%)	53 (9%)	9 (4%)
Physician did not provide information about the vaccine	161 (6%)	69 (12%)	7 (4%)
Do not want to be vaccinated	616 (21%)	74 (13%)	89 (44%)
No medical condition for which vaccination is recommended	–	118 (20%)	–
Other reasons	195 (7%)	16 (3%)	16 (8%)

## Footnotes

1 World Health Organization (2025). Vaccines and immunization [online]. Website: https://www.who.int/health-topics/vaccines-and-immunization. [accessed 20 Aug 2025].

2 Türkiye EKMUD (2024) Erişkin Bağışıklama Rehberi [online]. Website: https://view.publitas.com/yazilim-parki/eriskin-bagisiklama-rehbri-2024/page/1 [accessed 20 Aug 2025].

3 World Health Organization (2021). Immunization Agenda 2030 [online]. Website: https://www.who.int/teams/immunization-vaccines-and-biologicals/strategies/ia2030 [accessed 20 Aug 2025].
